# The Time-Dependent Effect of Assistance on Peritoneal Dialysis Duration: An Analysis of Data from the French Language Peritoneal Dialysis Registry

**DOI:** 10.34067/KID.0000000577

**Published:** 2024-09-19

**Authors:** Annabel Boyer, Antoine Lanot, Maxence Ficheux, Sonia Guillouet, Clémence Bechade, Thierry Lobbedez

**Affiliations:** 1 Centre Universitaire des Maladies Rénales, CHU de Caen, Caen, France; 2 U1086 INSERM – ANTICIPE – Centre Régional de Lutte Contre le Cancer, François Baclesse, Caen, France; 3 Normandie Université, Unicaen, UFR de Médecine, Caen, France

**Keywords:** epidemiology and outcomes, peritoneal dialysis

## Abstract

**Key Points:**

- It is unknown whether the benefit of assisted peritoneal dialysis (PD) programs appears immediately after PD initiation or rather after some time spent on PD.
- The protective effect of assisted PD on the risk of transfer to hemodialysis was not constant over time; it started after the first 6 months on PD.
- Assisted PD programs should be sustainable for at least 6 months to observe their benefits.

**Background:**

Peritoneal dialysis (PD) patient compliance is crucial for the prevention of complications. Assistance is associated with a lower risk of transfer to hemodialysis. As the risk of noncompliance increases over time, the protective effect of assistance on the risk of transfer to hemodialysis may not be immediate after PD initiation, but rather may appear after some time on PD. We aimed to analyze the time-varying effect of assistance on the risk of PD cessation.

**Methods:**

This retrospective study was conducted using data from the French Language PD Registry of incident PD patients between 2002 and 2018. Because of nonproportional hazards, with a change in the effect of the assistance modality on the different outcomes appearing at 6 months after PD initiation, the associations between the assistance modality and the different outcomes were explored using time-dependent coefficient Cox regression.

**Results:**

The study included 15,675 patients; 6717 deaths, 4973 transfers to hemodialysis, and 3065 kidney transplantations occurred. Both patients receiving nurse- and family-assisted PD had a lower risk of transfer to hemodialysis (mean cause-specific hazard ratio [cs-HR], 0.67; 95% confidence interval [CI], 0.62 to 0.72; and mean cs-HR, 0.75; 95% CI, 0.67 to 0.84). In the first 6 months after PD initiation, nurse-assisted PD patients had a greater risk of transfer to hemodialysis (<6 months cs-HR, 1.18; 95% CI, 1.03 to 1.36) but had a lower likelihood afterward (≥6 months cs-HR, 0.57; 95% CI, 0.53 to 0.62). Family-assisted PD was not associated with the risk of transfer to hemodialysis in the first 6 months after PD initiation, and those patients had a lower risk of transfer to hemodialysis afterward (≥6 months cs-HR, 0.72; 95% CI, 0.63 to 0.82).

**Conclusions:**

When implementing a national nurse-assisted PD program, its positive impact on PD duration should not be expected immediately after PD initiation. Assisted PD programs should be sustainable for at least 6 months to observe their benefits.

**Podcast:**

This article contains a podcast at https://www.asn-online.org/media/podcast/K360/2024_10_31_KID0000000577.mp3

## Visual Abstract

**Figure:**
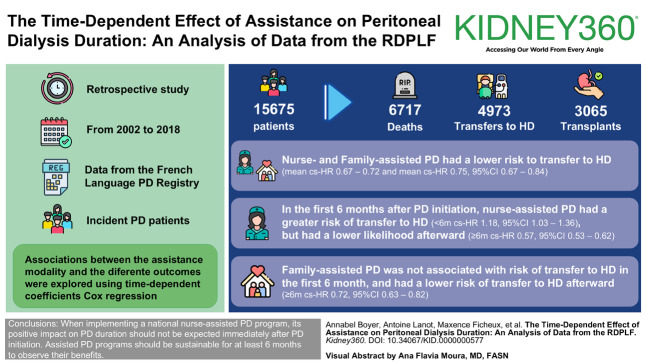

## Introduction

With peritoneal dialysis (PD) being a home-based therapy, patient compliance is crucial for preventing complications.^1^ Noncompliance and deviation from protocols are well-established issues; indeed, at 6 months after PD initiation, self-PD patients are more likely to be noncompliant than patients who are dependent on someone else to perform their dialysis.^2^ Furthermore, patient burnout and/or disease burden could affect the outcome of patients on home dialysis.

Assistance programs have been implemented in several countries in recent decades to further expand PD access to older and frailer populations.^3–5^ France has one of the longest experiences of assisted PD.^5,6^ According to previous studies, compared with self-care PD patients, nurse-assisted PD patients have a lower risk of being transferred to hemodialysis.^7^ Nurse assistance is also known to help prevent peritonitis in diabetic and elderly patients.^8,9^

More recently, the development of assisted PD programs in the United States has emerged. A recent pilot study in which nurses provided temporary support to enable patients to gain independence from staff assistance within 90 days was shown to be feasible.^10^ Short-term assistance may increase PD uptake at dialysis initiation; however, the positive effect on PD duration is questionable because the need for assistance may increase with the time spent on PD. As the risk of noncompliance increases over time,^2^ one may hypothesize that the protective effect of assistance on the risk of transfer to hemodialysis is not immediate after PD initiation, but rather appears after a period spent on PD. The time-varying protective effect of assistance may also depend on the cause of PD cessation.

This study aimed to analyze the time-varying effect of nurse and family assistance on the risk of PD cessation. The secondary objectives were to analyze the time-varying cause-specific effect of assistance on the risk of transfer to hemodialysis.

## Methods

### Study Population

This retrospective observational study was performed using data from the French Language PD Registry (French Language Peritoneal Dialysis Registry [RDPLF]). All adults older than 18 years who started PD in France between January 1, 2002, and December 31, 2018, were included. The end of the study period was December 31, 2021.

### Definition of Variables

Age at PD onset, sex, underlying nephropathy, diabetes mellitus status, previous therapy before PD initiation, PD modality at dialysis initiation (continuous ambulatory PD or automated PD), registration on a waiting list for kidney transplantation, suboptimal starters (defined as a period of <30 days on hemodialysis before PD initiation),^11^ center category, and the use of assistance were obtained from the registry. The Charlson Comorbidity Index was extracted from the database to evaluate patient comorbidities, and the modified Charlson Comorbidity Index was calculated by subtracting the age subscore. For the center size estimation, we calculated the number of incident PD patients per center per year of participation during the study period.

### Explanatory Variables

The main explanatory variable was the modality of assisted PD: nurse-assisted PD or family-assisted PD. Self-care PD was used as a comparator.

### Events of Interest

PD cessation was the outcome of interest, studied by the occurrence of death on PD or transfer to hemodialysis for more than 2 months (composite end point), death on PD (transfer to hemodialysis–censored) and transfer to hemodialysis (death-censored) for more than 2 months.^12^ Transplantation was considered a censoring event.

For the secondary objectives, the events of interest were the different causes of transfer to hemodialysis (infection, inadequate dialysis [inadequate solute clearance associated with uremic syndrome or inadequate ultrafiltration associated with overhydration, or malnutrition], PD catheter issues, social issues, other causes linked with PD, and other causes not linked with PD), which were studied one at a time. Death, renal transplantation, and other causes of transfer to hemodialysis were considered censoring events.

### Statistical Analysis

Categorical variables are described by their absolute numbers and percentages, while continuous variables are described by their medians and interquartile ranges. Peritonitis episodes per patient-year were reported, as recommended by the International Society for PD.

In an etiological approach, cause-specific hazard ratios (cs-HRs) and their 95% confidence intervals (CIs) were calculated using a Cox regression model to explore the association between assistance modality and the events of interest. All variables considered potentially relevant *a priori* were included in the multivariate models.

Cox regression was used to analyze time-to-event data. We consider the general hazard model, where h0(*t*) is the baseline hazard function and *β*′ is a vector of regression coefficients. In the usual form of Cox regression, X is a vector of time-fixed covariates H(*t*|X)=exp(*β*×X)×h0(*t*), in which the proportional hazard assumption is precisely that *β*(*t*)=*β*, that is, the coefficient, does not change over time, meaning that the effect of the covariate on the outcome cannot change over time. In the case of nonproportional hazards, time-dependent coefficients can be used with the following equation: H(*t*|X)=exp(*β*[*t*]×X)×h0(*t*).^13,14^ This approach allows us to obtain the exposure of the mean cs-HR and the cs-HR for separate time periods that can be selected based on the graphical representation of the coefficient value over time. It allows us to report the change of the effect of the covariate on the outcome over time.

**Figure 1:**
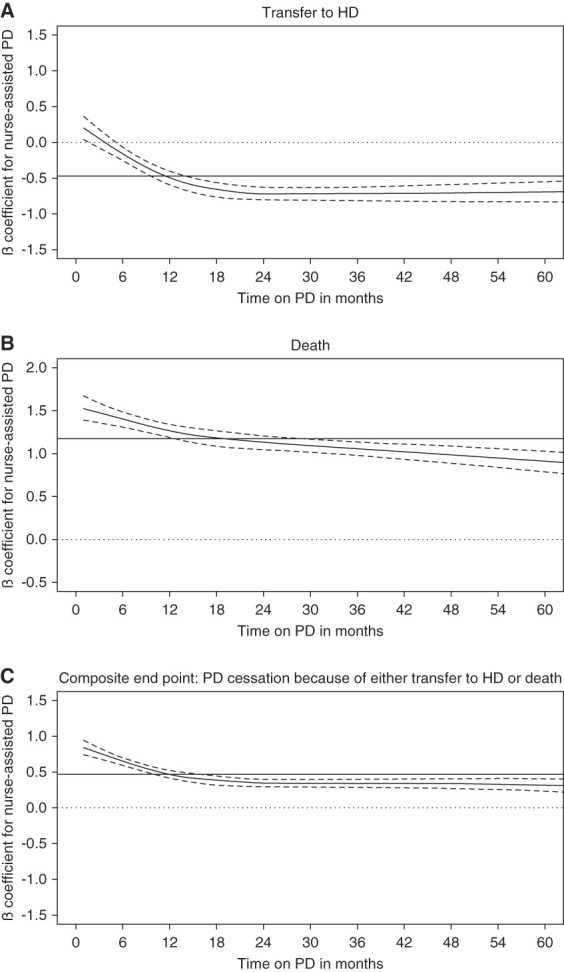
**Spline regressions estimating the baseline hazard of the effect of nurse assistance on the different outcomes of interest.** Spline plain line: evolution of the *β* coefficient; spline dotted lines: evolution of the 95% CIs; straight plain line: mean cs-HR. (A) Transfer to hemodialysis. (B) Death. (C) Composite end point: PD cessation because of either transfer to hemodialysis or death. CIs, confidence interval; cs-HR, cause-specific hazard ratio; HD, hemodialysis; PD, peritoneal dialysis.

Schoenfeld residual were statistically significant (*P* < 0.001), indicating that the effect of assistance significantly varies over time. The regression splines, an alternative for Schoenfeld residual plotting, that were used to estimate the baseline hazard of the effect of nurse assistance on the different outcomes of interest showed a change in the effect appearing around the sixth month after PD initiation (Figure 1). In an intention-to-treat approach, an adjusted Cox regression with time-dependent coefficients was thus used, as recommended by Therneau *et al*.^13^ As early PD cessation has been defined as a transfer to hemodialysis occurring within the first 6 months on PD^15^ and based on the aspect of the regression spline, a cutoff period at 6 months after PD initiation was arbitrarily selected, enabling us to estimate the adjusted hazard ratios of the effect of assistance on the different outcomes for the first 6 months of PD (<6 months cs-HR) and for those above 6 months after PD initiation (≥6 months cs-HR).

We performed sensitivity analysis by running the different time-dependent coefficient analyses with 12-, 18-, and 24-month cutoff periods and by running the different survival models on the subset of patients new to PD, excluding patients with a history of hemodialysis before PD.

Less than 2% missing data were noted, which we assumed were missing at random, enabling us to perform a complete case analysis. Statistical analyses were performed with R version 4.0.2 (R Foundation for Statistical Computing, Vienna, Austria).

The RDPLF has the approval of the French National Ethics Committee (*Commission Nationale de l’Informatique et des Libertés*). This study took place within the framework of this authorization. This study was reported in accordance with the Strengthening the Reporting of Observational Studies in Epidemiology guidelines (2008).

## Results

### Patient Characteristics

Of the 15,675 incident patients on PD included in the study, 6809 (44%) and 1316 (8%) were on nurse-assisted PD and family-assisted PD, respectively. Compared with self-care PD patients, nurse-assisted and family-assisted PD were older and had more comorbidities. Self-care PD patients were more frequently registered on the waiting list for kidney transplantation (Table 1).

**Table 1:** Patient characteristics according to the modality of assistance

Characteristic	All Patients (*n*=15,675)	Nurse-Assisted (*n*=6809)	Family-Assisted (*n*=1316)	Self-Care (*n*=7550)
Age at PD initiation, yr, median (IQR)	70.4 (55.8–80.2)	79.6 (72.8–84.5)	73.9 (65.8–80.9)	57.3 (44.4–68.4)
Sex (male), *No.* (%)	9431 (60.2)	3707 (54.4)	824 (52.6)	4900 (64.9)
Diabetes, *No.* (%)	5163 (32.9)	2906 (42.7)	585 (44.5)	1672 (22.1)
**Modified CCI, *No.* (%)**				
2	4875 (31.1)	1064 (15.6)	209 (15.9)	3602 (47.7)
3	2905 (18.5)	1312 (19.3)	231 (17.5)	1362 (18)
4	2557 (16.3)	1349 (19.8)	223 (16.9)	985 (13.1)
≥5	4968 (31.7)	2922 (42.9)	610 (46.4)	1436 (19)
Missing	370 (2.4)	162 (2.4)	43 (3.3)	165 (2.2)
**Underlying nephropathy, *No.* (%)**				
Diabetic	3047 (19.4)	1658 (24.4)	348 (26.4)	1041 (13.8)
GN	2365 (15.1)	417 (6.1)	107 (8.1)	1841 (24.4)
Interstitial nephritis	941 (6)	332 (4.9)	72 (5.5)	537 (7.1)
Polycystic kidney disease	1001 (6.4)	144 (2.1)	43 (3.3)	814 (10.8)
Systemic disease	417 (2.7)	105 (1.5)	25 (1.9)	287 (3.8)
Unknown	1818 (11.6)	869 (12.8)	144 (10.9)	805 (10.7)
Uropathy	507 (3.2)	118 (1.7)	35 (2.7)	354 (4.7)
Vascular	4981 (31.8)	2965 (43.5)	477 (36.3)	1539 (20.4)
Other	459 (2.9)	138 (2)	46 (3.5)	275 (3.6)
Missing	139 (0.9)	63 (1)	19 (1.4)	57 (0.7)
**PD start year, *No.* (%)**				
2002–2007	4873 (31.1)	2117 (31.1)	503 (38.2)	2253 (29.9)
2007–2013	4244 (27.1)	1841 (27)	393 (29.9)	2010 (26.6)
2013–2018	6558 (41.8)	2851 (41.9)	420 (31.9)	3287 (43.5)
Suboptimal starters, *n* (%)	1423 (9.1)	692 (10.2)	114 (8.7)	617 (8.2)
**Treatment before PD, *No.* (%)**				
No therapy	12,557 (80.1)	5628 (82.7)	1058 (80.4)	5871 (77.8)
Hemodialysis	2637 (16.8)	1130 (16.6)	236 (17.9)	1271 (16.8)
Renal transplantation	481 (3.1)	51 (0.7)	22 (1.7)	408 (5.4)
First PD modality (CAPD), *No.* (%)	12,358 (78.8)	6164 (90.5)	1031 (78.3)	5163 (68.4)
Awaiting renal transplantation	4139 (26.4)	255 (3.7)	125 (9.5)	3759 (49.8)
**Type of center, *No.* (%)**				
Community hospital	7586 (48.4)	3391 (49.8)	675 (51.3)	3520 (46.6)
Academic hospital	3064 (19.5)	1210 (17.8)	259 (19.7)	1595 (21.1)
Nonprofit	3677 (23.5)	1549 (22.7)	266 (20.2)	1862 (24.7)
Private hospital	1338 (8.5)	654 (9.6)	115 (8.7)	569 (7.5)
Missing	10 (0.1)	5 (0.1)	1 (0.1)	4 (0.1)
**Center experience, *No.* (%) (new patients per yr)**				
≤10	11,382 (72.6)	4838 (71.1)	1032 (78.4)	5512 (73)
>10	4293 (27.4)	1971 (28.9)	284 (21.6)	2038 (27)

CAPD, continuous ambulatory peritoneal dialysis; CCI, Charlson Comorbidity Index; IQR, interquartile range; PD, peritoneal dialysis.

### Outcomes on PD

Over the study period, 6717 (43%) patients died, 4973 (32%) transferred to hemodialysis, and 3065 (20%) underwent kidney transplantation. Among the 7550 self-care PD patients, 3014 (40%) were transferred to hemodialysis, 1214 (16%) died, and 2827 (37%) underwent kidney transplantation. Among the 6809 patients on nurse-assisted PD, 4733 (70%) died, 1577 (23%) transferred to hemodialysis, and 149 (2%) underwent kidney transplantation.

Of the 15.675 patients, 5870 (37%) had at least one peritonitis event during the study period: self-care PD: 2955 (39%) events, family-assisted PD: 495 (38%) events, nurse-assisted PD: 2420 (35%) events. The peritonitis rate was 0.36/patient year in the self-care PD group, 0.37/patient-year in the family-assisted PD group, and 0.32/patient-year in the nurse-assisted PD group.

Among the 4973 patients who were transferred to hemodialysis, the causes of transfer were inadequate dialysis (1995), infection (815), mechanical issues (417), social issues (424), other causes linked to PD (559), and other causes not linked to PD (753).

### PD Cessation by Death or Transfer to Hemodialysis (Transplantation-Censored)

According to the usual adjusted Cox regressions, compared with self-care PD patients, both nurse- and family-assisted PD patients had a greater risk of PD cessation (mean cs-HR, 1.19; 95% CI, 1.13 to 1.25; and mean cs-HR, 1.18; 95% CI, 1.10 to 1.27; respectively) (Table 2). According to the adjusted Cox regressions with time-dependent coefficients, both nurse- and family-assisted PD patients had greater risks of PD cessation in the first 6 months after PD initiation (<6 months cs-HR, 1.70; 95% CI, 1.54 to 1.88 and cs-HR, <1.40; 95% CI, 1.20 to 1.64; respectively) and to a lesser extent above 6 months after PD initiation (≥6 months cs-HR, 1.10; 95% CI, 1.04; to 1.16 and cs-HR, 1.15; 95% CI, 1.06 to 1.24; respectively).

**Table 2:** Association of the effect of the assistance modality on the different outcomes, adjusted for patient characteristics in a multivariate Cox regression analysis

Explanatory Variable	Transfer to Hemodialysis, *n*=4973, cs-HR (95% CI)	Death, *n*=6717, cs-HR (95% CI)	Death or Transfer to Hemodialysis, *n*=11,690, cs-HR (95% CI)
**Assistance status**			
Self	Reference	Reference	Reference
Nurse	0.67 (0.62 to 0.72)	1.98 (1.85 to 2.14)	1.19 (1.13 to 1.25)
Family	0.75 (0.67 to 0.84)	1.97 (1.79 to 2.17)	1.18 (1.10 to 1.27)
**Age at PD initiation**			
<65	Reference	Reference	Reference
65–80	0.64 (0.59 to 0.68)	1.26 (1.16 to 1.38)	0.83 (0.79 to 0.88)
≥80	0.45 (0.41 to 0.50)	1.99 (1.81 to 2.18)	1.07 (1.01 to 1.14)
Sex, female	0.84 (0.79 to 0.90)	0.88 (0.83 to 0.92)	0.87 (0.83 to 0.90)
Diabetes	1.11 (1.02 to 1.22)	0.98 (0.92 to 1.05)	1.03 (0.98 to 1.09)
**Modified CCI**			
2	Reference	Reference	Reference
3	0.85 (0.78 to 0.93)	1.32 (1.21 to 1.45)	1.02 (0.97 to 1.09)
4	0.85 (0.77 to 0.92)	1.63 (1.49 to 1.78)	1.16 (1.09 to 1.23)
≥5	0.80 (0.73 to 0.87)	2.03 (1.86 to 2.21)	1.32 (1.24 to 1.40)
**Underlying nephropathy**			
Diabetes	Reference	Reference	Reference
Interstitial nephritis	0.94 (0.81 to 1.10)	0.92 (0.81 to 1.06)	0.94 (0.85 to 1.04)
GN	1.09 (0.97 to 1.23)	0.92 (0.82 to 1.04)	1.09 (1.01 to 1.18)
Unknown	0.96 (0.84 to 1.09)	1.20 (1.08 to 1.32)	1.09 (1.01 to 1.17)
Uropathy	0.88 (0.73 to 1.06)	0.76 (0.62 to 0.95)	0.85 (0.74 to 0.98)
Vascular	0.89 (0.80 to 0.99)	1.16 (1.07 to 1.25)	1.06 (0.99 to 1.13)
Systemic disease	1.13 (0.93 to 1.36)	1.11 (0.89 to 1.39)	1.14 (0.99 to 1.31)
Other	0.76 (0.62 to 0.93)	1.23 (1.04 to 1.46)	0.98 (0.86 to 1.11)
Polycystic kidney disease	1.02 (0.88 to 1.19)	0.93 (0.77 to 1.12)	1.05 (0.94 to 1.17)
**PD start year**			
2002–2007	Reference	Reference	Reference
2007–2013	1.17 (1.09 to 1.27)	0.92 (0.86 to 0.98)	1.01 (0.96 to 1.06)
2013–2018	1.42 (1.32 to 1.52)	1.03 (0.97 to 1.10)	1.16 (1.11 to 1.22)
**Therapy before PD initiation**			
No therapy	Reference	Reference	Reference
Hemodialysis	1.19 (1.10 to 1.28)	1.13 (1.06 to 1.21)	1.16 (1.10 to 1.22)
Transplantation	1.51 (1.31 to 1.73)	1.18 (0.92 to 1.53)	1.44 (1.28 to 1.63)
Suboptimal starters	0.97 (0.87 to 1.08)	1.07 (0.98 to 1.17)	1.04 (0.97 to 1.11)
**First PD modality**			
CAPD	Reference	Reference	Reference
APD	1.08 (1.01 to 1.16)	0.85 (0.78 to 0.92)	0.97 (0.92 to 1.02)
Awaiting renal transplantation	0.39 (0.36 to 0.42)	0.20 (0.17 to 0.24)	0.38 (0.35 to 0.41)
**Type of center**			
Academic hospital	Reference	Reference	Reference
Community hospital	0.91 (0.84 to 0.99)	1.03 (0.96 to 1.11)	0.98 (0.93 to 1.03)
Nonprofit	0.99 (0.91 to 1.09)	0.93 (0.86 to 1.01)	0.96 (0.90 to 1.02)
Private hospital	1.16 (1.03 to 1.31)	1.12 (1.01 to 1.25)	1.14 (1.06 to 1.24)
**Center experience (new patients per year)**			
≤10	Reference	Reference	Reference
>10	0.86 (0.80 to 0.92)	1.09 (1.03 to 1.16)	0.99 (0.95 to 1.04)

APD, automated peritoneal dialysis; CAPD, continuous ambulatory peritoneal dialysis; CCI, Charlson Comorbidity Index; CI, confidence interval; cs-HR, cause-specific hazard ratio; PD, peritoneal dialysis.

### PD Cessation by Death (Transfer to Hemodialysis and Transplantation-Censored)

**Figure 2:**
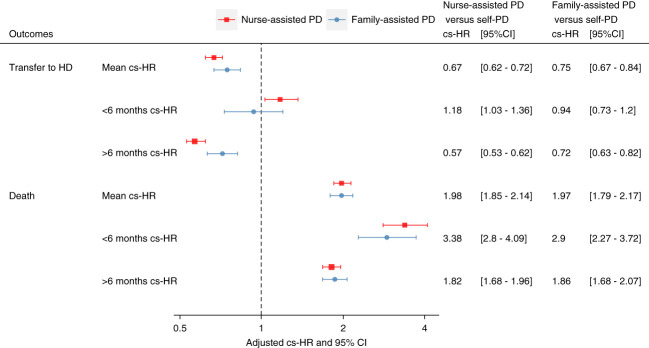
**Forest plot representing the effect of the assistance modality on the risks of transfer to hemodialysis and death in the usual adjusted Cox regression and adjusted Cox regression analyses with time-dependent coefficients.** On the basis of the aspect of the regression spline, a cutoff period at 6 months after PD initiation was selected, enabling us to estimate the adjusted hazard ratios of the effect of assistance on the different outcomes for the first 6 months of PD (<6 months cs-HR) and for those above 6 months after PD initiation (≥6 months cs-HR). HR, hazard ratio.

Regardless of the modality and timing, assisted PD was associated with a greater risk of death: mean cs-HR, 1.98; 95% CI, 1.85 to 2.14; <6 months cs-HR, 3.38; 95% CI, 2.80 to 4.09; and ≥6 months cs-HR, 1.85; 95% CI, 1.68 to 1.96 for nurse assistance; and mean cs-HR, 1.97; 95% CI, 1.79 to 2.17; <6 months cs-HR, 2.90; 95% CI, 2.27 to 3.72; and ≥6 months cs-HR, 1.86; 95% CI, 1.68 to 2.07 for family assistance (Figure 2).

### PD Cessation by Transfer to Hemodialysis (Death and Transplantation-Censored)

Compared with self-care PD patients, both nurse- and family-assisted PD patients had a lower risk for transfer to hemodialysis (mean cs-HR, 0.67; 95% CI, 0.62 to 0.72; and mean cs-HR, 0.75; 95% CI, 0.67 to 0.84; respectively) (Figure 2 and Table 2).

In the first 6 months after PD initiation, patients on nurse-assisted PD had a greater risk of transfer to hemodialysis (<6 months cs-HR, 1.18; 95% CI, 1.03 to 1.36) but a lower likelihood afterward (≥6 months cs-HR, 0.57; 95% CI, 0.53 to 0.62). Family-assisted PD was not associated with the risk of transfer to hemodialysis in the first 6 months after PD initiation (<6 months cs-HR, 0.94; 95% CI, 0.73 to 1.20), but those patients had a lower risk of transfer to hemodialysis afterward (≥6 months cs-HR, 0.72; 95% CI, 0.63 to 0.82) (Figure 2).

### Cause-Specific Analysis of Transfer to Hemodialysis

#### Transfer to Hemodialysis for Inadequate Dialysis

**Figure 3:**
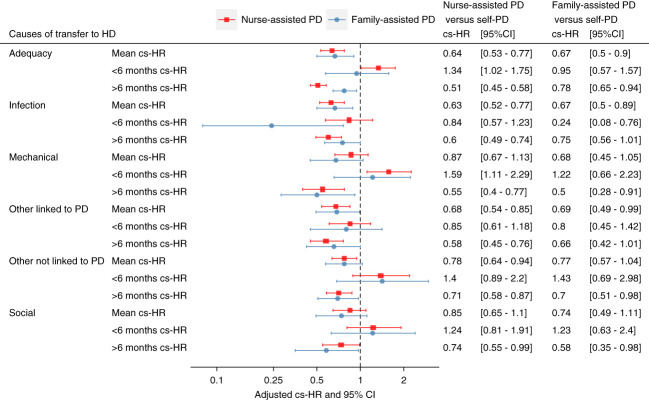
**Forest plot representing the effect of the assistance modality on the risks of the different causes of transfer to hemodialysis according to the usual adjusted Cox regression and adjusted Cox regression analyses with time-dependent coefficients.** On the basis of the aspect of the regression spline, a cutoff period at 6 months after PD initiation was selected, enabling us to estimate the adjusted hazard ratios of the effect of assistance on the different outcomes for the first 6 months of PD (<6 months cs-HR) and for those above 6 months after PD initiation (≥6 months cs-HR).

In the first 6 months on PD, nurse assistance was associated with a greater risk of transfer to hemodialysis because of inadequate dialysis (<6 months cs-HR, 1.34; 95% CI, 1.02 to 1.75), while the risk decreased afterward (≥6 months cs-HR, 0.51; 95% CI, 0.45 to 0.58). Family assistance was not associated with the risk of transfer to hemodialysis because of inadequate dialysis in the first 6 months after PD initiation (<6 months cs-HR, 0.95; 95% CI, 0.57 to 1.57), and the risk decreased afterward (≥6 months cs-HR, 0.78; 95% CI, 0.65 to 0.94) (Figure 3).

#### Transfer to Hemodialysis for Infection

During the first 6 months on PD, nurse assistance was not associated with the risk of transfer to hemodialysis because of infection; however, a lower risk was observed after 6 months on PD (≥6 months cs-HR, 0.60; 95% CI, 0.49 to 0.74). By contrast, family-assisted PD was strongly associated with a decreased risk of transfer because of infection in the first 6 months after PD initiation (<6 months cs-HR, 0.24; 95% CI, 0.08 to 0.76), but it was not associated with this outcome afterward (≥6 months cs-HR, 0.75; 95% CI, 0.56 to 1.01) (Figure 3).

#### Transfer to Hemodialysis for Catheter-Related Problems

In the first 6 months after PD initiation, nurse-assisted PD patients had a greater risk of transfer to hemodialysis because of catheter-related problems (<6 months cs-HR, 1.59; 95% CI, 1.11 to 2.29); family-assisted PD was not associated with this outcome (<6 months cs-HR, 1.22; 95% CI, 0.66 to 2.23). After the first 6 months on PD, both assistance modalities were associated with a lower risk of transfer to hemodialysis because of catheter-related problems (Figure 3).

#### Transfer to Hemodialysis for Social Issues, Other Causes Linked to PD, and Other Causes Not Linked to PD

The associations between the assisted PD modality and the risk of transfer because of social issues, other causes linked to PD, and other causes not linked to PD are described in Figure 3.

### Sensitivity Analyses

The analyses performed defining the cutoff period at 12 months provided similar results (Supplemental Figure 1). However, when defining the cutoff period at either 18 or 24 months, both nurse and family assistance were protective against the risk of transfer to hemodialysis in both time periods (before and after 18 months and before and after 24 months) (Supplemental Figures 2 and 3).

The analyses performed on the subset of patients experiencing new to PD, excluding patients without a history of hemodialysis before PD, provided similar results (Supplemental Figure 4).

## Discussion

Our work showed that patients treated with nurse- and family-assisted PD had a greater risk of death and a lower risk of transfer to hemodialysis, which is consistent with the results of previous studies from our team.^7,16,17^ However, the protective effect of assisted PD on the risk of transfer to hemodialysis, independent of the assistance modality, was not constant over time; it started between the first 6–12 months on PD. Notably, nurse assistance was protective against all causes of transfer to hemodialysis after the first 6–12 months of PD, but the association was stronger for transfers for inadequate dialysis, catheter-related problems, and infection. We know that short-term assistance is associated with PD uptake.^18^ However, our results suggest that one should not expect to observe the positive impact of assisted PD programs on the PD duration immediately after PD initiation, but rather that assisted PD programs need to be sustainable for a long period, at least 6 months, to observe their benefit.

Noncompliance with PD treatment in self-care PD patients is a well-identified issue; indeed, up to 35% of PD patients are reported to be noncompliant.^19^ Noncompliance often occurs in the first 6 months of PD. In addition, noncompliant patients had worse outcomes on PD and, notably, a higher rate of transfer to hemodialysis,^2^ maybe because of adequacy issues as a result of regularly missing one or two PD dwell a day. Although the effect of assistance may vary over time for a variety of reasons, not specifically noncompliance and burnout, it could be hypothesized that assistance might improve compliance with the prescription and consequently decrease the risk of transfer to hemodialysis because of adequacy issues.

PD life participation is one of the core outcomes for patients on PD reported by the standardised outcomes in nephrology-peritoneal dialysis initiative.^20^ The burnout of PD patients and their caregivers, because of the burden of the disease, has been identified as a significant contributor to PD cessation and transfer to hemodialysis.^21,22^ Social isolation has been described as one of the burdens of PD, with patients facing the disease alone experiencing an intensified feeling of exhaustion.^23,24^ The International Society for PD guidelines recommend routinely assessing the impact of dialysis treatment on life participation.^25^ In situations of psychological exhaustion and isolation, nurse-assisted PD could serve as a respite for either the patient or caregivers to prevent burnout.^23^ The reduced risk of transfer, notably for inadequate dialysis and social issues, of patients on nurse-assisted PD could reflect a decreased risk of burnout in the assisted PD population.

In France, nurse-assisted PD is fully covered by French health care insurance, with a private nurse visiting the patient at home to perform PD. According to the RDPLF, >50% of incident patients treated with PD needed assistance.^26^ A previous study by our team described a linear decline in family assistance over the past decade, with assisted PD relying mainly on nurse assistance.^27^ In January 2024, 527,153 nurses were registered with a valid license of nursing practice, 19% of whom practiced in the private sector.^28^ With an aging population and an increase in home therapies, the workload of private nurses is constantly increasing.^29^ The nurse population is expected to grow between 37% and 61% by 2040 in the face of needs, which are expected to increase by at least 54%.^28,30^

Nurse-assisted PD was associated with a greater risk of transfer to hemodialysis in the first 6 months after PD initiation, especially for transfers because of catheter-related problems (Figure 3). An explanation could be that nurses are witnessing first hand the consequences of mechanical catheter problems or that nurses are (mis)attributing ongoing deterioration in frailty or symptoms in comorbid patients as due to adequacy issues, advocating for a transfer to hemodialysis more effectively than the patient or family would have done.

Compared with self-care PD, the peritonitis rates were higher in the family-assisted PD group and lower in the nurse-assisted PD group, meaning that nurse assistance could reduce the risk of transfer to hemodialysis partially through reduced peritonitis. Interestingly, family-assisted PD was protective against the risk of transfer because of infection in the first 6 months after PD initiation, but this protective effect did not remain significant after this period. Protocol deviation is known to increase with the time spent on PD,^1^ and the loss of protection from family assistance on the risk of transfer for infection after 6 months of PD could reflect an increased number of breaches during aseptic procedures. The impact of the disease burden on family caregivers may also explain this finding. Our results highlight the importance of assessing the involvement of family helpers during treatment.

Our study has limitations. By nature, the observational design cannot lead to conclusions regarding causality. The retrospective nature of any registry study is associated with possible classification bias. The causes of PD cessation were declarative in the registry, which could have led to declaration bias. Residual confounders, such as residual kidney function, membrane characteristics and detailed information on PD prescription, and educational programs, are optionally declared in the registry and thus not fully captured, although these factors are known to affect technique survival. We lacked the data regarding the possible change in assistance status. However, our study was conceived with an intention-to-treat approach, meaning that we decided to analyze the assistance status and not the assistance that was eventually received nor the possible changes of assistance over time. In addition, although mortality has been shown to be higher in the first month after transfer from PD to hemodialysis,^31^ we lacked the data of events occurring after transfers to hemodialysis. The cutoff period at 6 months after PD initiation was arbitrarily defined, on the basis of the regression spline. Our findings appear to be robust in that the numerous sensitivity analyses performed provided similar results.

Finally, as the use of nurse assistance is largely conditional on national health care organizations and funding, the generalizability of our results to countries other than France may not be appropriate.

In conclusion, our study shows that in France, both nurse- and family-assisted PD are associated with lower risks of transfer to hemodialysis, especially because of lower risks of transfer for dialysis adequacy and infection. However, the protective effect of assisted PD on the risk of transfer to hemodialysis, independent of the assistance modality, appears between the first 6–12 months on PD. Our results suggest that when implementing a national nurse-assisted PD program, the positive impact of assisted PD programs on the PD duration should not be expected immediately after PD initiation; rather, assisted PD programs need to be sustainable for a period of at least 6–12 months to observe their benefits.

## Supplementary Material

SUPPLEMENTARY MATERIAL

## Data Availability

Partial restrictions to the data and/or materials apply. Data will be shared on request, registry data.

## Supplemental Material

Supplemental Figure 1. Forest plot representing the effect of the assistance modality on the risks of transfer to hemodialysis and death in the usual adjusted Cox regression and adjusted Cox regression analyses with time-dependent coefficients, with a cutoff period at 12 months.

Supplemental Figure 2. Forest plot representing the effect of the assistance modality on the risks of transfer to hemodialysis and death in the usual adjusted Cox regression and adjusted Cox regression analyses with time-dependent coefficients, with a cutoff period at 18 months.

Supplemental Figure 3. Forest plot representing the effect of the assistance modality on the risks of transfer to hemodialysis and death in the usual adjusted Cox regression and adjusted Cox regression analyses with time-dependent coefficients, with a cutoff period at 24 months.

Supplemental Figure 4. Forest plot representing the effect of the assistance modality on the risks of transfer to hemodialysis and death in the usual adjusted Cox regression and adjusted Cox regression analyses with time-dependent coefficients, with a cutoff period at 6 months, on the subset of patients new to PD (excluding patients with a history of hemodialysis before PD).
