# Supplementary material for: The Time-Dependent Effect of Assistance on Peritoneal Dialysis Duration: An Analysis of Data from the French Language Peritoneal Dialysis Registry
Source: Kidney360. 2024 Sep 19;5(10):1500–9. doi: 10.34067/KID.0000000577 (PMC11556925; doi:10.34067/KID.0000000577)
Supplement: SUPPLEMENTARY MATERIAL [file kidney360-5-1500-s001.pdf]

ASN Journal Disclosure Form

As per ASN journal policy, I have disclosed any financial relationships or commitments I have held in the past 36 months as included below. I have listed my Current Employer below to indicate there is a relationship requiring disclosure. If no relationship exists, my Current Employer is not listed.

C. Béchade reports the following:
Employer: CHU de Caen; Normandie Université; Consultancy: -Baxter; -Fresenius; Honoraria: Baxter, Fresenius, Astellas; and Speakers Bureau: Baxter, Fresenius.

I understand that the information above will be published within the journal article, if accepted, and that failure to comply and/or to accurately and completely report the potential financial conflicts of interest could lead to the following: 1) Prior to publication, article rejection, or 2) Post-publication, sanctions ranging from, but not limited to, issuing a correction, reporting the inaccurate information to the authors' institution, banning authors from submitting work to ASN journals for varying lengths of time, and/or retraction of the published work.

Name: Clémence Béchade
Manuscript ID: K360-2024-000380R1
Manuscript Title: The time-dependent effect of assistance on peritoneal dialysis duration: an analysis of data from the RDPLF
Date of Completion: July 5, 2024
Disclosure Updated Date: July 5, 2024

ASN Journal Disclosure Form

As per ASN journal policy, I have disclosed any financial relationships or commitments I have held in the past 36 months as included below. I have listed my Current Employer below to indicate there is a relationship requiring disclosure. If no relationship exists, my Current Employer is not listed.

A. Boyer reports the following:
Honoraria: Baxter

I understand that the information above will be published within the journal article, if accepted, and that failure to comply and/or to accurately and completely report the potential financial conflicts of interest could lead to the following: 1) Prior to publication, article rejection, or 2) Post-publication, sanctions ranging from, but not limited to, issuing a correction, reporting the inaccurate information to the authors' institution, banning authors from submitting work to ASN journals for varying lengths of time, and/or retraction of the published work.

Name: Annabel Boyer
Manuscript ID: K360-2024-000380R1
Manuscript Title: The time-dependent effect of assistance on peritoneal dialysis duration: an analysis of data from the RDPLF
Date of Completion: July 8, 2024
Disclosure Updated Date: July 8, 2024

ASN
American Society of Nephrology
JASN
Journal of the American Society of Nephrology
KIDNEY360
Accessing Our World From Every Angle
CJASN
Clinical Journal of the American Society of Nephrology

ASN Journal Disclosure Form

As per ASN journal policy, I have disclosed any financial relationships or commitments I have held in the past 36 months as included below. I have listed my Current Employer below to indicate there is a relationship requiring disclosure. If no relationship exists, my Current Employer is not listed.

M. Ficheux reports the following:
Employer: CHU Caen; and Consultancy: Fresenius medical care;.

I understand that the information above will be published within the journal article, if accepted, and that failure to comply and/or to accurately and completely report the potential financial conflicts of interest could lead to the following: 1) Prior to publication, article rejection, or 2) Post-publication, sanctions ranging from, but not limited to, issuing a correction, reporting the inaccurate information to the authors' institution, banning authors from submitting work to ASN journals for varying lengths of time, and/or retraction of the published work.

Name: Maxence Ficheux
Manuscript ID: K360-2024-000380R2
Manuscript Title: The time-dependent effect of assistance on peritoneal dialysis duration: an analysis of data from the RDPLF
Date of Completion: September 7, 2024
Disclosure Updated Date: September 7, 2024

ASN American Society of Nephrology | JASN Journal of the American Society of Nephrology | KIDNEY360 Accessing Our World From Every Angle | CJASN Clinical Journal of the American Society of Nephrology

ASN Journal Disclosure Form

As per ASN journal policy, I have disclosed any financial relationships or commitments I have held in the past 36 months as included below. I have listed my Current Employer below to indicate there is a relationship requiring disclosure. If no relationship exists, my Current Employer is not listed.

S. Guillouët has nothing to disclose.

I understand that the information above will be published within the journal article, if accepted, and that failure to comply and/or to accurately and completely report the potential financial conflicts of interest could lead to the following: 1) Prior to publication, article rejection, or 2) Post-publication, sanctions ranging from, but not limited to, issuing a correction, reporting the inaccurate information to the authors' institution, banning authors from submitting work to ASN journals for varying lengths of time, and/or retraction of the published work.

Name: Sonia Guillouët
Manuscript ID: K360-2024-000380R2
Manuscript Title: The time-dependent effect of assistance on peritoneal dialysis duration: an analysis of data from the RDPLF
Date of Completion: August 21, 2024
Disclosure Updated Date: August 21, 2024

ASN American Society of Nephrology
JASN Journal of the American Society of Nephrology
KIDNEY360 Accessing Our World From Every Angle
CJASN Clinical Journal of the American Society of Nephrology

ASN Journal Disclosure Form

As per ASN journal policy, I have disclosed any financial relationships or commitments I have held in the past 36 months as included below. I have listed my Current Employer below to indicate there is a relationship requiring disclosure. If no relationship exists, my Current Employer is not listed.

A. Lanot reports the following:
Employer: Caen University Hospital; Consultancy: CSL Vifor; Bayer Health; AstraZeneca; and Honoraria: CSL Vifor; Baxter; Fresenius; Bayer Health; AstraZeneca.

I understand that the information above will be published within the journal article, if accepted, and that failure to comply and/or to accurately and completely report the potential financial conflicts of interest could lead to the following: 1) Prior to publication, article rejection, or 2) Post-publication, sanctions ranging from, but not limited to, issuing a correction, reporting the inaccurate information to the authors' institution, banning authors from submitting work to ASN journals for varying lengths of time, and/or retraction of the published work.

Name: Antoine Lanot
Manuscript ID: K360-2024-000380R2
Manuscript Title: The time-dependent effect of assistance on peritoneal dialysis duration: an analysis of data from the RDPLF
Date of Completion: September 4, 2024
Disclosure Updated Date: August 26, 2024

ASN Journal Disclosure Form

As per ASN journal policy, I have disclosed any financial relationships or commitments I have held in the past 36 months as included below. I have listed my Current Employer below to indicate there is a relationship requiring disclosure. If no relationship exists, my Current Employer is not listed.

T. Lobbedez reports the following:
Employer: NEPHROLOGY CHU CAEN; Consultancy: ASTELLAS; Honoraria: ASTELLAS; and Patents or Royalties: ASTELLAS.

I understand that the information above will be published within the journal article, if accepted, and that failure to comply and/or to accurately and completely report the potential financial conflicts of interest could lead to the following: 1) Prior to publication, article rejection, or 2) Post-publication, sanctions ranging from, but not limited to, issuing a correction, reporting the inaccurate information to the authors' institution, banning authors from submitting work to ASN journals for varying lengths of time, and/or retraction of the published work.

Name: Thierry Lobbedez
Manuscript ID: K360-2024-000380R2
Manuscript Title: "The time-dependent effect of assistance on peritoneal dialysis duration: an analysis of data from the RDPLF"
Date of Completion: July 25, 2024
Disclosure Updated Date: July 25, 2024
