# Supplementary material for: The Time-Dependent Effect of Assistance on Peritoneal Dialysis Duration: An Analysis of Data from the French Language Peritoneal Dialysis Registry
Source: Kidney360. 2024 Sep 19;5(10):1500–9. doi: 10.34067/KID.0000000577 (PMC11556925; doi:10.34067/KID.0000000577)
Supplement: SUPPLEMENTARY MATERIAL [file kidney360-5-1500-s002.pdf]

Supplementary data

Supplemental Figure 1. Forest plot representing the effect of the assistance modality on the risks of transfer to HD and death in the usual adjusted Cox regression and adjusted Cox regression analyses with time-dependent coefficients, with a cutoff period at 12 months.

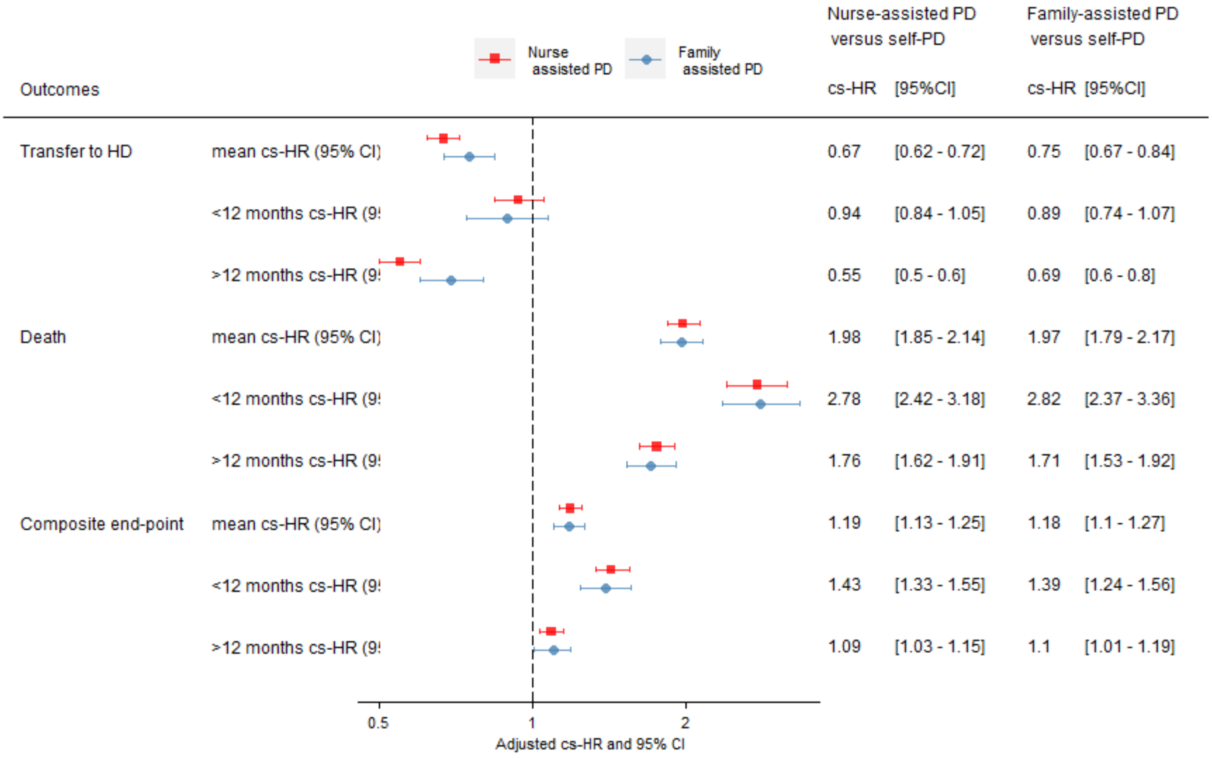

HD: hemodialysis; cs-HR: cause-specific hazard ratio; 95% CI: 95% confidence interval

A cutoff period at 12 months after PD initiation was selected, enabling us to estimate the adjusted hazard ratios of the effect of assistance on the different outcomes for the first 12 months of PD (<12 months cs-HR) and for those above 12 months after PD initiation (≥12 months cs-HR).

Supplemental Figure 2. Forest plot representing the effect of the assistance modality on the risks of transfer to HD and death in the usual adjusted Cox regression and adjusted Cox regression analyses with time-dependent coefficients, with a cutoff period at 18 months.

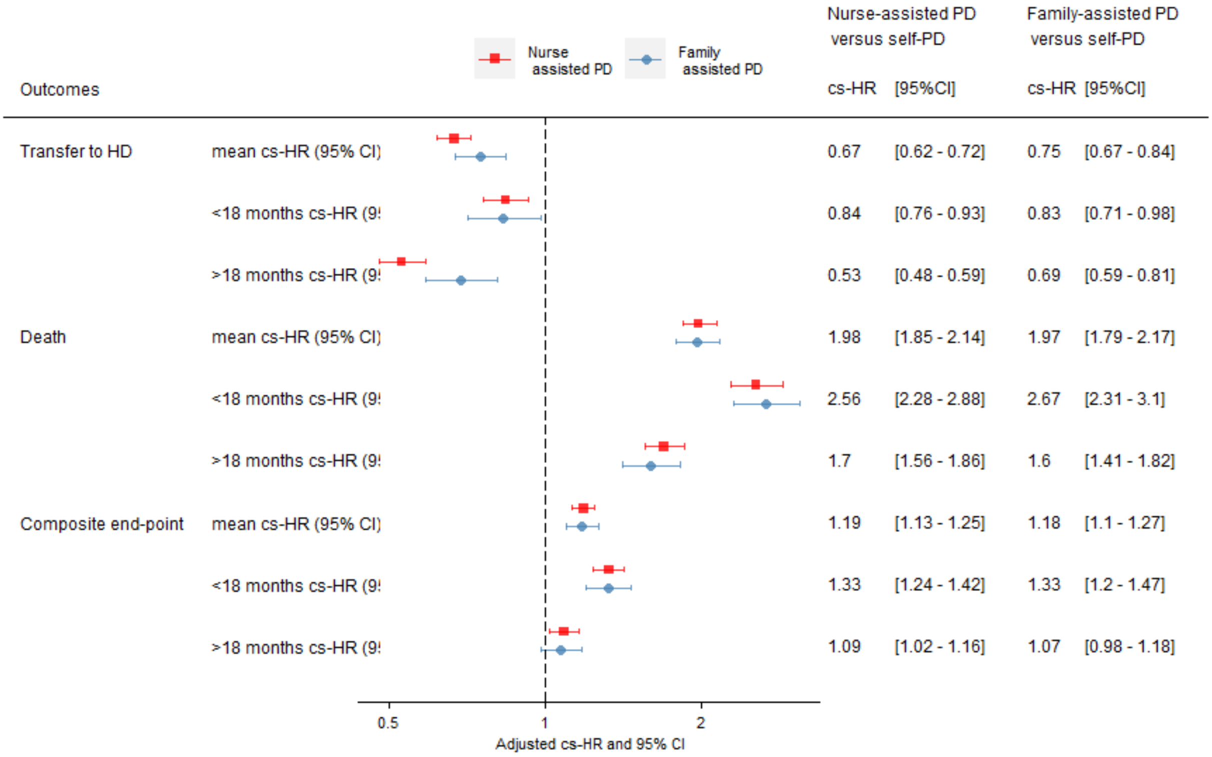

HD: hemodialysis; cs-HR: cause-specific hazard ratio; 95% CI: 95% confidence interval

A cutoff period at 18 months after PD initiation was selected, enabling us to estimate the adjusted hazard ratios of the effect of assistance on the different outcomes for the first 18 months of PD (<18 months cs-HR) and for those above 18 months after PD initiation (≥18 months cs-HR).

Supplemental Figure 3. Forest plot representing the effect of the assistance modality on the risks of transfer to HD and death in the usual adjusted Cox regression and adjusted Cox regression analyses with time-dependent coefficients, with a cutoff period at 24 months.

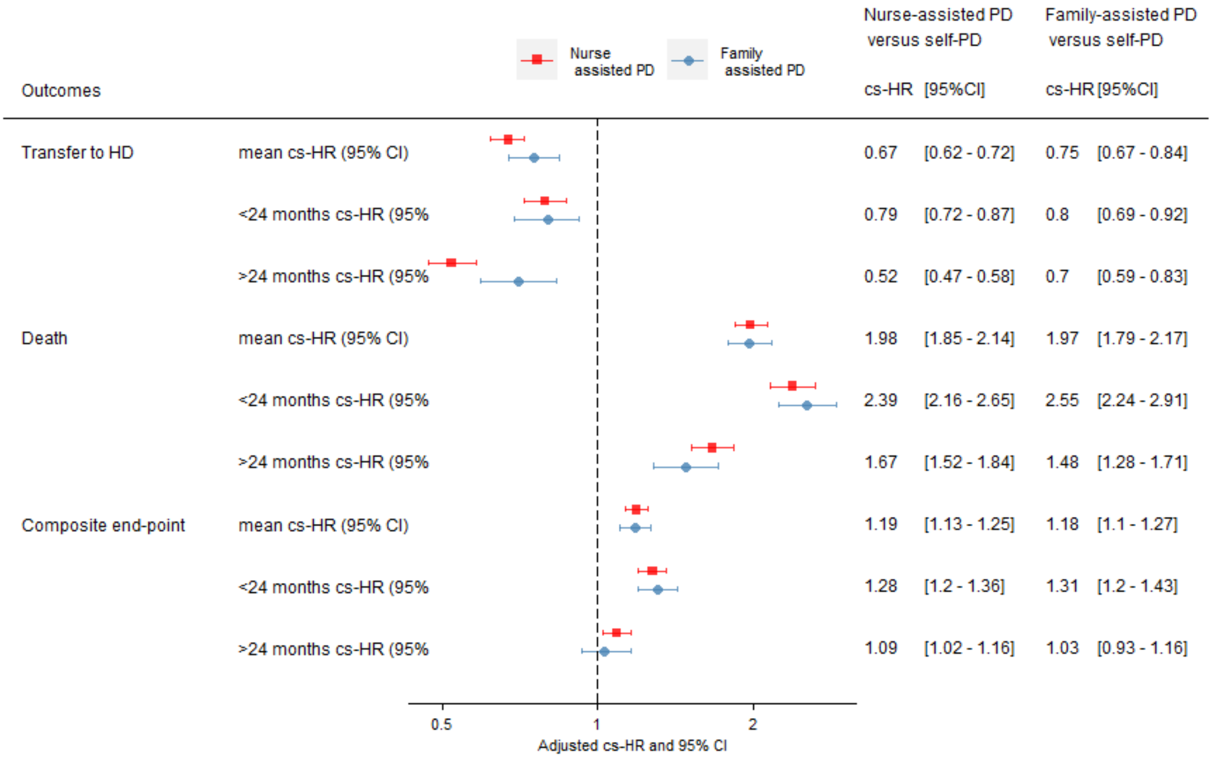

HD: hemodialysis; cs-HR: cause-specific hazard ratio; 95% CI: 95% confidence interval

A cutoff period at 24 months after PD initiation was selected, enabling us to estimate the adjusted hazard ratios of the effect of assistance on the different outcomes for the first 24 months of PD (<24 months cs-HR) and for those above 24 months after PD initiation (≥24 months cs-HR)

Supplemental Figure 4. Forest plot representing the effect of the assistance modality on the risks of transfer to HD and death in the usual adjusted Cox regression and adjusted Cox regression analyses with time-dependent coefficients, with a cutoff period at 6 months, on the subset of patients new to PD (excluding patients with a history of HD prior to PD).

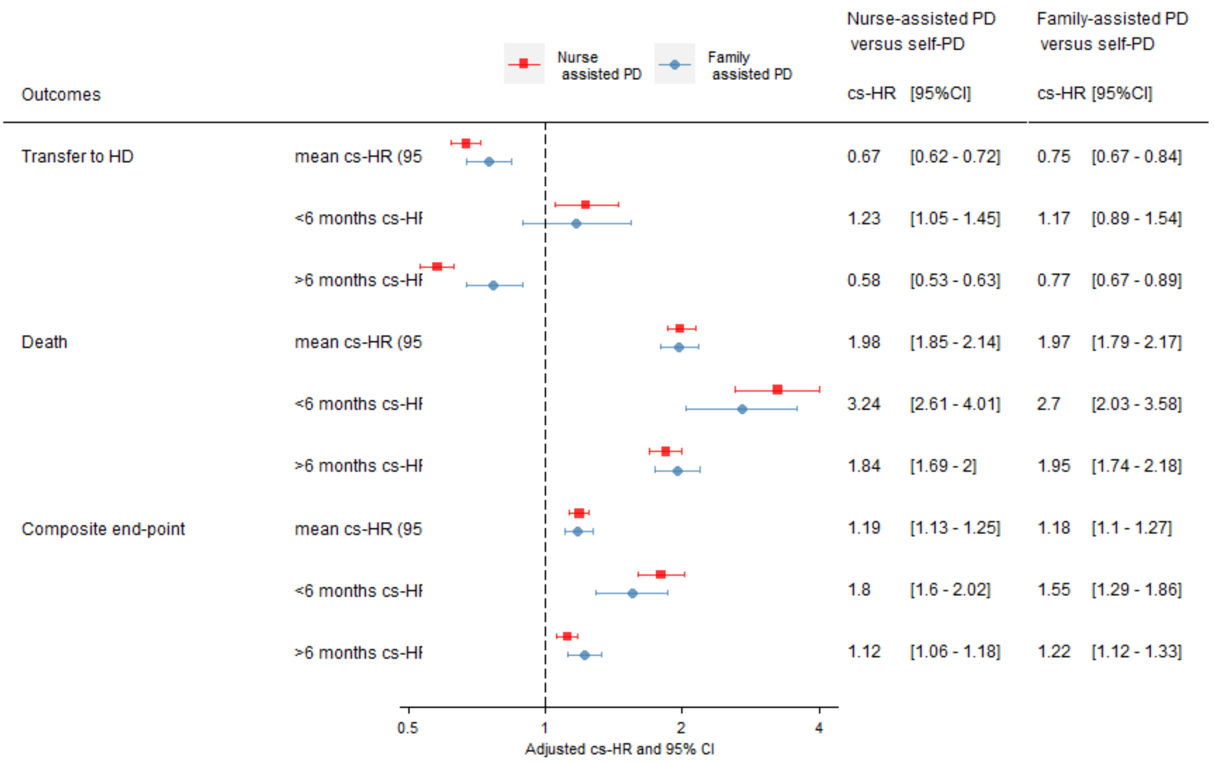

HD: hemodialysis; cs-HR: cause-specific hazard ratio; 95% CI: 95% confidence interval

A cutoff period at 6 months after PD initiation was selected, enabling us to estimate the adjusted hazard ratios of the effect of assistance on the different outcomes for the first 6 months of PD (<6 months cs-HR) and for those above 6 months after PD initiation (≥6 months cs-HR)
